# Editorial for the Special Issue on Particles Separation in Microfluidic Devices, Volume II

**DOI:** 10.3390/mi13030482

**Published:** 2022-03-20

**Authors:** Naotomo Tottori, Takasi Nisisako

**Affiliations:** 1 Department of Mechanical Engineering, Faculty of Engineering, Kyushu University, W4-729, 744, Motooka, Nishi-ku, Fukuoka 819-0395, Japan; 2 Institute of Innovative Research, Tokyo Institute of Technology, R2-9, 4259 Nagatsuta-cho, Midori-ku, Yokohama 226-8503, Japan

Particle separation in the nano- to microscale range is a significant step for biological, chemical, and medical analyses. Since the 2000s, many micro- and nanofluidic techniques have been developed for the separation, sorting, isolation, fractionation, and purification of various particles, especially for biological particles, based on their chemical and/or physical properties. These micro- and nanofluidic techniques have been attracting attention as a promising approach because they enable us to use a sample and reagent with minimum consumption, are easy to use, and can be integrated into other components for comprehensive analysis. These micro- and nanofluidic techniques are classified into two types; (i) passive techniques using the hydrodynamic effect induced by the geometries in the micro/nanoscale, and (ii) active techniques using external forces, for example in the magnetic, optical, acoustic, and electric fields.

The papers collected in this Special Issue present state-of-the-art research for particle separation and manipulation using microfluidic techniques. There are five research papers and a review article published in this Special Issue. Three research papers present the separation and manipulation of particles by measn of the passive approach using layers of stacked beads in a microchannel [1], three-dimensional (3D) channels [2], and convex air bubbles attached to the surface of the channel [3]. The remaining papers present the separation of particles by means of the integration of active and passive techniques using an electric field and a deterministic lateral displacement (DLD) array [4,5]. The review article covers microfluidic technologies for separation and detection toward achieving practical applications for public health [6].

(1) Passive method: Xu et al. proposed a microfluidic plasma separator for biochemical analysis using three layers of different-sized microspheres as the separation structure [1]. They designed a microfluidic device with 18 capillary microchannels and enabled the extraction of ~3 μL of plasma from a 50 μL blood sample in ~55 min. As a demonstration of the feasibility of the device in the application of clinical biochemical testing, they introduced a clinical blood sample into the device and measured the concentrations of four components (TP, ALB, GLU, and UA), and obtained measurement values similar to those provided by conventional centrifugal separation, indicating acceptable accuracy for point-of-care analysis. Zoupanou et al. reported a method for the fabrication of a 3D fluidic mixer made from poly(methylmethacrylate) (PMMA) using computer-aided design (CAD) and the micromilling technique and demonstrated the manipulation of the fluid and nanoparticles [2]. They performed passive chaotic mixing and dilution through the reservoir and serpentine layers in the fabricated device. Park et al. proposed a novel technique to prevent the clogging of microspheres in the channel of the catheter by utilizing convex air bubbles attached to the surface of the channel walls [3]. In this paper, they evaluated the effect of the width, cavity, and the distances between the cavities to prevent the clogging of microspheres. From the experimental results, they established that the large convex air bubbles with a small distance between the two adjacent cavities can effectively prevent the clogging of microspheres in the channel.
(2) Integration of the passive and active methods: Ho et al. reported the separation of particles with nano- and micro-sizes primarily based on their zeta potential by combining DLD methods with electric fields (eDLD) [4]. In this study, they performed the characterization of the relevant parameters (e.g., ionic strength, applied voltage, frequency, and pressure) necessary to achieve adequate separation of particles with different types, enabling them to adapt the method to various particle sizes and zeta potentials. They also demonstrated the separation of nano-sized liposomes with different lipid components with biological relevance. Ho et al. also reported the separation of cells based on their differences in the membrane and/or internal structure by combining a DLD technique with electrokinetics [5]. Using the proposed microfluidic device, they performed the separation of cells, which were heat-treated to deactivate cells for changing their viability and structure, based on their viability (viable or non-viable cells). For the separation of *Escherichia coli* (*E. coli*), the change in their size after deactivation is not sufficient for the size-based separation of the DLD array; however, they utilized the considerable change in their zeta potential, and achieved separation of *E. coli* by applying AC voltage in the DLD array at a low frequency. In contrast, for the separation of *Saccharomyces cerevisiae* (Baker’s yeast), the change in zeta potential after heat treatment is small, and therefore they utilized the change in dielectrophoretic property and achieved the separation of Baker’s yeast by applying AC voltage in the DLD array at a higher frequency.

In addition to these research articles, Zhang et al. presented a comprehensive review of the latest developments of microfluidic separation and detection for the benefits of public health. These two topics (i.e., separation and detection) are normally reviewed separately; however, they are related closely to each other for their application to public health, and understanding the techniques of separation will offer new insights to develop detection technologies.

We would like to thank all authors who submitted their papers to this Special Issue. We would also like to acknowledge all of the reviewers who dedicated their time to provide their reports in a timely manner in order to improve the quality of the papers collated in this Special Issue. 
